# Comparative efficacy of combined and single neuromuscular electrical stimulation and traditional swallowing training for neurogenic dysphagia: a network meta-analysis

**DOI:** 10.3389/fneur.2025.1700317

**Published:** 2025-12-03

**Authors:** Shuxuan Wang, Zhenyu Shi, Tingting Wu, Chong Li, Chuchu Huang, Di Sun

**Affiliations:** 1 Tongde Hospital of Zhejiang Province Affiliated to Zhejiang Chinese Medical University (College of Integrated Traditional Chinese and Western Medicine Clinical Medicine), Hangzhou, Zhejiang, China; 2 Hangzhou First People's Hospital Xiasha Campus, Hangzhou, Zhejiang, China; 3 Tongde Hospital of Zhejiang Province, Hangzhou, Zhejiang, China

**Keywords:** dysphagia, neuromuscular electrical stimulation, traditional swallowing training, network meta-analysis, neurorehabilitation

## Abstract

**Objective:**

This study aims to compare the efficacy of combined and single neuromuscular electrical stimulation (NMES), and traditional swallowing training (TST) for neurogenic dysphagia through a network meta-analysis (NMA).

**Methods:**

This meta-analysis has been prospectively registered on the PROSPERO (Registration number: CRD42025643351). Electronic databases, including Embase, PubMed, Web of science and the Cochrane Library, were searched up to March 1, 2025. All published randomized controlled trials (RCTs) comparing combined and/or single neuromuscular electrical stimulation with traditional swallowing therapy for the treatment of patients with neurogenic dysphagia were included. A network meta-analysis using STATA software synthesized data and ranked treatments by efficacy. The outcome measures included the Functional Oral Intake Scale (FOIS), Standardized Swallowing Assessment (SSA), Penetration-Aspiration Scale (PAS) and clinical efficacy (CE).

**Results:**

Twenty-two RCTs with a total sample size of 1,265 cases were ultimately included. The findings suggest that NMES combined with other therapies is more effective than single NMES or traditional swallowing therapy for patients with neurogenic dysphagia, demonstrating a higher clinical efficacy rate. Among the combined therapies, the integration of NMES with transcranial direct current stimulation (tDCS) and TST demonstrated the highest efficacy in improving FOIS scores and enhancing swallowing function [surface under cumulative ranking curve values (SUCRCV): 95.3%, standardized mean difference (SMD): 1.15, 95% confidence interval (CI): 0.34, to 1.97]. Additionally, NMES combined with acupuncture showed the most significant reduction in SSA scores (SUCRA: 87.6%, SMD: −1.49, 95% CI: −2.48 to −0.49). Furthermore, the combination of NMES combined with effortful swallowing (ES) and TST exhibited the most pronounced effects in lowering PAS scores and preventing aspiration (SUCRA: 81.2%, SMD: −1.06, 95% CI: −1.82 to −0.31).

**Conclusions:**

Ranking probabilities indicated that combined therapy had the highest likelihood of being the most effective intervention. However, large-scale, multi-center, high-quality studies are essential to further validate this conclusion.

## 1 Introduction

Neurogenic dysphagia, a prevalent complication of neurological disorders such as stroke, traumatic brain injury, and neurodegenerative diseases, significantly impairs patients' quality of life and increases the risk of aspiration pneumonia (1). Restoration of swallowing function in patients with neurogenic dysphagia depends on effective management strategies and preventing life-threatening complications. Neuromuscular electrical stimulation (NMES) has emerged as a promising adjunctive therapy for neurogenic dysphagia. NMES delivers electrical stimulation to muscles via surface electrodes, inducing muscle contraction through depolarization of nerve fibers, thereby enhancing swallowing muscle strength and sensory awareness to improve or restore swallowing function (2, 3). Traditional swallowing therapy, which includes exercises to strengthen swallowing muscles and improve swallowing mechanisms, remains the cornerstone of dysphagia rehabilitation. However, the relative efficacy of single NMES therapy, combined NMES therapy, and traditional swallowing therapy remains unclear. Although a comprehensive meta-analysis has confirmed that NMES combined with swallowing training is superior to swallowing training alone in improving swallowing function and reducing complications (4), this finding does not elucidate the comparative effectiveness of other NMES-based combination therapies. Moreover, the optimal pairing of NMES with other modalities, such as transcranial direct current stimulation (tDCS), acupuncture, or effortful swallowing, has not been systematically ranked.

Therefore, this study aims to compare the efficacy of combined and single NMES therapies with traditional swallowing therapy alone in the treatment of neurogenic dysphagia through a network meta-analysis, with the goal of providing evidence for optimizing treatment strategies for neurogenic dysphagia in clinical practice.

## 2 Methods

### 2.1 Protocol registration

This meta-analysis has been prospectively registered on the PROSPERO (Registration number: CRD42025643351). This systematic review adhered to the PRISMA-2020 guidelines.

### 2.2 Ethics

Since this treatment does not entail the recruitment of clients or the collection of personal info, ethical clearance is not essential.

### 2.3 Search strategy

We searched electronic databases, including Embase, PubMed, Web of Science, and the Cochrane Library, from their inception to March 1, 2025. The search was limited to studies published in English and Chinese. A combination of Medical Subject Headings (MeSH) terms and free-text words was employed. The search terms included: “dysphagia”, “Swallowing Disorders”, “Oropharyngeal Dysphagia”, “neuromuscular electrical stimulation”, “NMES”, “electrostimulation”, “randomized controlled trial”, “RCT”, and their related synonyms and variants in both English and Chinese. The detailed search strategy is illustrated in Supplementary Table 1.

### 2.4 Inclusion criteria

Literature search and testing procedures were conducted independently by two researchers to determine eligibility. Any differences in the results were discussed and resolved through consensus. Originally, titles and abstracts were screened to recognize pertinent studies. Ultimately, an in-depth examination of full texts was conducted to identify whether each research study satisfied the adhering to criteria:

(1) Literature type: studies were included if they were explicitly described as randomized controlled trials (RCTs), regardless of whether blinding was employed.
(2) Participants: ① Individuals aged 18 years or older. ② Patients diagnosed with neurogenic dysphagia secondary to neurological conditions, such as stroke, traumatic brain injury, Parkinson's disease, or multiple sclerosis. To address potential differences among these neurological conditions, only patients with clear diagnostic criteria and comparable treatment goals were included, ensuring the homogeneity and comparability of participants and reducing the impact of disease-specific variability on the results.
(3) Interventions: combined treatment group: neuromuscular electrical stimulation (NMES) combined with other therapies. Single treatment group: NMES alone or traditional swallowing therapy alone.
(4) Outcome indicators: the study included at least one of the following outcome measures: Functional oral intake scale (FOIS), standardized swallowing assessment (SSA), penetration-aspiration scale (PAS) and clinical efficacy. For the outcome of CE, it was defined as a significant improvement or recovery in swallowing function as per the original authors' criteria in each respective study, typically measured by a predefined clinically important improvement on standardized scales such as FOIS, SSA or a global clinician assessment as reported.

### 2.5 Exclusion criteria

(1) Studies published in languages other than English or Chinese.
(2) Studies involving children or adolescents (aged <18 years).
(3) Studies focusing on non-neurogenic dysphagia caused by conditions such as esophageal tumors or scleroderma.
(4) Animal studies, review articles, case reports, gray literature, dissertations, conference abstracts or studies other than RCTs.
(5) Studies with interventions or outcome measures that do not meet the inclusion criteria, or those with flawed experimental design or methodology.
(6) Studies with incomplete data or unavailable full-text articles.

### 2.6 Quality of literature

Two authors independently assessed the risk of bias using the Cochrane tool. Inter-rater reliability was evaluated with Cohen's kappa coefficient. The two scientists evaluated the literary works's efficiency on assessment criteria and made judgments of low, uncertain, and high danger, respectively, and then cross-checked them. If there were any kind of discrepancies, we would certainly review them. If agreement could not be gotten to, we would certainly review it with a third party scientist.

### 2.7 Interpretation of league tables

In a network meta-analysis, the league table is used to present the relative effectiveness of different interventions. The rows and columns represent various treatments, and the intersecting cells display the pairwise effect estimates along with their 95% confidence intervals. In this study, if a confidence interval does not include the null value (such as an odds ratio of 1 or a mean difference of 0), the comparison is considered statistically significant and is indicated accordingly in the table. The reported effect size reflects the relative efficacy of the treatment in the row compared to the treatment in the column.

### 2.8 Statistical analysis

The frequentist method used random effect models was employed to conduct network meta-analyses (NMAs) of randomized controlled trials. A common heterogeneity parameter was assumed in the network meta-analysis aimed to compare with the empirical distributions of heterogeneity. An assessment of global and local statistical heterogeneity was conducted with generalized Cochran's Q. Continuous variables (FOIS, PAS, SSA) were expressed as standardized mean differences (SMD) with their 95% confidence intervals (CI), while dichotomous variables (clinical effective rate) were expressed as risk ratios (RR) with their 95% CI. For studies involving three or more arms, the research will be split and error-corrected before inclusion in the website meta-analysis. Since control groups are prone to repeated use, their sample sizes will be proportionally reduced based on the number of splits and comparisons. To evaluate transitivity, we examined the distributions of key study characteristics across comparison groups; any detected differences were explored via meta-regression to assess their impact on the results. We assessed statistical inconsistency between direct and indirect evidence using both global and local tests.

As global approach, we used a design-by-treatment interaction model to investigate the inconsistency in the entire network. We evaluated local inconsistency based on the node-splitting method. We produced league tables and calculated the surface under the cumulative ranking curve (SUCRA) for each intervention. Publication bias was assessed using comparison-adjusted funnel plots. Regarding missing or incomplete data, studies with essential missing outcome data that could not be retrieved through author contact or calculated from available statistics were excluded during the full-text screening phase to ensure the integrity of the analysis. Statistical analyses were conducted using the network package based on Stata MP16.0 (StataCorp LP, College Station, TX, USA) and RStudio software (R Foundation for Statistical Computing). A significance level of two-sided α = 0.05 was set for all analyses.

## 3 Results

### 3.1 Study selection

**Figure 1:**
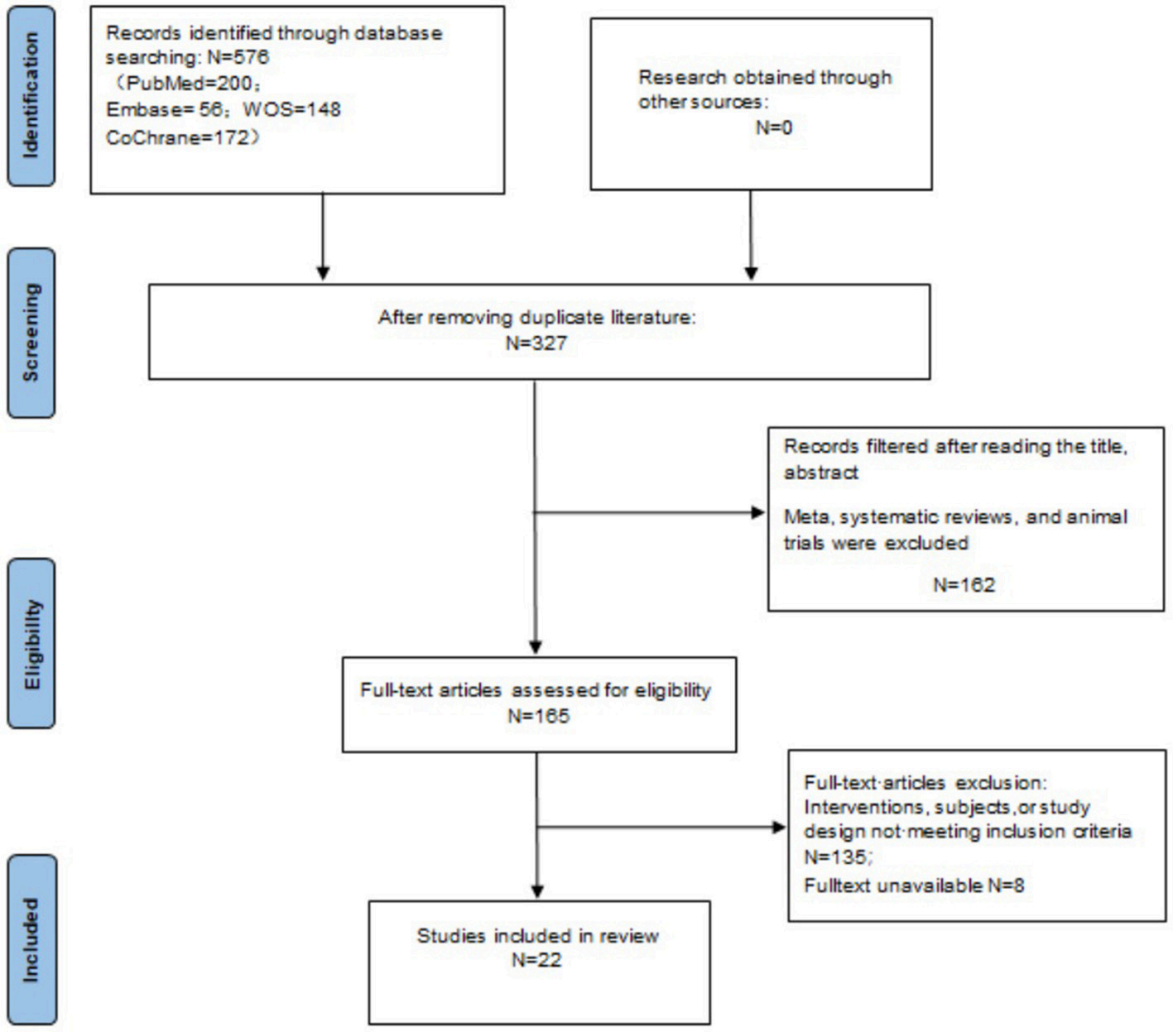
Flowchart of study screening process.

A total of 576 records were retrieved from the following databases: PubMed (*n* = 200), Web of Science (*n* = 148), Embase (*n* = 56), and the Cochrane Library (*n* = 172). After removing 249 duplicate records, a stepwise screening process was conducted, resulting in the inclusion of 22 studies (2, 5–20). The detailed screening process is illustrated in Figure 1.

### 3.2 Study characteristics

In the selected 22 studies, the total sample size was 1,265 cases, and a total of 10 intervention measures were investigated, including neuromuscular electrical stimulation (abbreviated as NMES), traditional swallowing training (abbreviated as TST), neuromuscular electrical stimulation combined with traditional swallowing training (abbreviated as NMES+TST), neuromuscular electrical stimulation combined with transcranial direct current stimulation and traditional swallowing training (abbreviated as NMES+tDCS+TST), neuromuscular electrical stimulation combined with acupuncture (abbreviated as NMES+AP), neuromuscular electrical stimulation combined with upper cervical spine mobilization (abbreviated as NMES+UCSM), neuromuscular electrical stimulation combined with acupoint injection (abbreviated as NMES+AI), neuromuscular electrical stimulation combined with effortful swallowing and traditional swallowing training (abbreviated as NMES+ES+TST), neuromuscular electrical stimulation combined with eating training and traditional swallowing training (abbreviated as NMES+ET+TST), neuromuscular electrical stimulation combined with Swallowing Strengthening Exercises (abbreviated as NMES+SSE). Detailed characteristics of the included studies are presented in Table 1.

**Table 1:** Basic characteristics of the included trials.

**Study ID**	**Age, yr M/F**		**Sample size M/F**		**Duration of illness**		**Intervention**		**Disease**	**Treatment period**	**Outcome measure**
**N**	**T**	**C**	**T**	**C**	**T**	**C**	**T**	**C**		**T/C**	**T/C**
Huang 2014	64.5 ± 14.4	67.0 ± 10.1	11	8	16.6 ± 6.6 days	25.2 ± 16.0 days	NMES	TST	Stroke	3 weeks	FOIS, VFSS, PAS, FDS
	68.9 ± 9.8		10		23.5 ± 13.9 days		NMES+TST				
Lee 2021	68.9 ± 11.0	73.4 ± 6.7	26	23	44.9 ± 59.7 days	40.2 ± 38.4 days	NMES+TST	TST	Stroke, brain tumor, encephalitis	Immediately	VDS, PAS
Permsirivanich 2009	64.50 ± 8.80	64.73 ± 9.39	12	11	>2 weeks		NMES	TST	Stroke	4 weeks	FOIS
Bengisu 2024	68.0 ± 10.5	66.9 ± 12.5	10	10			NMES+TST	TST			
	65.2 ± 11.5	63.1 ± 14.2	10	10	3.1 ± 2.5 days		NMES+tDCS +TST	tDCS+TST	Stroke	2 weeks	GUSS, VFSS, PAS, FOIS, DSRS
Cakmak 2023	62.9 ± 9.8	63.6 ± 10.0	17	17	48.3 ± 92.6 weeks	52.2 ± 92.2 weeks	NMES+TST	TST	Stroke	3 weeks	FOIS, EAT-10, SWAL-QOL, VRQOL
Cao 2023	61 ± 8	59 ± 7	30	30	34.0 ± 22.1 days	36.8 ± 47.4 days	NMES+AP	NMES	Stroke	4 weeks	SSA, PAS, CE
Chang 2014	46 ± 10	44 ± 11	38	36	16.6 ± 4.8 days	17.3 ± 5.2 days	NMES+AP	NMES	Stroke	4 weeks	SDSS, NTRR, CE
Jeon 2020	63.12 ± 13.50	64.47 ± 8.43	17	17	6 months-2 years		NMES+UCSM	NMES	Stroke	4 weeks	CCFT, CVA, VDS, PAS
Li 2019	23.8 ± 6.28	23.6 ± 6.29	30	30			NMES+TST	TST	Wilson's disease	8 weeks	WST, SSA
Liang 2021	62.74 ± 6.14	63.14 ± 5.49	36	36	31.27 ± 5.85 days	31.85 ± 6.34 days	NMES+TST	TST	Stroke	4 weeks	CE, SSA, KDWT
	51.2 ± 10.8	47.5 ± 10.5	40	32	24.4 ± 7.1 days	23.8 ± 5.2 days	NMES	AI			
Ma 2014	50.6 ± 11.1	49.9 ± 11.8	35	40	25.3 ± 8.4 days	25.2 ± 6.9 days	NMES+AI	TST	Stroke	30 days	CE, WST
		51.5 ± 10.9	28			24.5 ± 5.7 days		Sham			
Ma 2015	66.8 ± 7.3	63.7 ± 8.4	40	40	25.7 ± 2.9 days	27.2 ± 1.9 days	NMES	AP	Stroke	2 weeks	CE, WST, SDSS
	66.5 ± 7.8		40		26.3 ± 2.6 days		NMES+AP				
Matos 2022	40-70		16	17	< 24 hours		NMES+TST	TST	Stroke	5 days	FOIS, FEES, DREP
Park 2018	63.44 ± 13.55	54.67 ± 13.82	9	9	36.44 ± 7.5 weeks	36.11 ± 7.94 weeks	NMES+ES+TST	TST	Parkinson's disease	4 weeks	VDS, PAS, VFSS
Park 2016	54.00 ± 11.93	55.80 ± 12.23	25	25	35.44 ± 5.63 weeks	36.00 ± 6.05 weeks	NMES+ES+TST	TST	Stroke	6 weeks	VDS, PAS, VFSS
Simonelli 2019	67.2 ± 16.2	72.4 ± 12.3	16	15	45.2 ± 22.3 days	32.6 ± 18.1 days	NMES+TST	TST	Stroke	8 weeks	FOIS, FEES, PAS, PS
Sproson 2018	76 ± 11.4	79 ± 11.4	12	12	9.1 ± 20.5 months	17.3 ± 25.0 months	NMES+SSE	TST	Stroke	4 weeks	FOIS, PAS, SWAL-QOL
Tarihci 2023	62.9 ± 9.8	63.6 ± 10.0	17	17	48.3 ± 92.6 weeks	52.2 ± 92.2 weeks	NMES+TST	TST	Stroke	3 weeks	FOIS, EAT-10, SWAL-QOL, VRQOL, FEES
Terre 2015	46 (28–60)	51 (22–69)	10	10	1-6 months		NMES+TST	TST	Stroke, brain injury	4 weeks	FOIS, OTT, PDT, PTT
Xia 2011	66.40 ± 15.63	65.32 ± 14.29	40	40	9.22 ± 3.88 days	8.94 ± 3.62 days	NMES	TST		
	65.85 ± 14.63		40		8.37 ± 3.12 days		NMES+TST				
									Stroke	4 weeks	sEMG, SSA, VFSS, SWAL-QOL
Zhang 2016	62.2 ± 9.2	62.6 ± 8.7	27	27	20.6 ± 4.3 days	21.3 ± 4.1 days	NMES+TST	TST	Stroke	4 weeks	WST, SSA, FOIS, SWAL-QOL, MMSE
Zhang 2021	63.72 ± 6.29	62.31 ± 8.07	27	28	2.10 ± 0.54 months	2.16 ± 0.50 months	NMES+ET+TST	TST	Stroke	6 weeks	CE, DOSS, SAP
	63.14 ± 6.56		28		2.06 ± 0.54 months		ET+TST				

T = treatment group, C = control group, M/F = male and female, yr = year, NMES = Neuromuscular Electrical Stimulation, TST = Traditional Swallowing Training, tDCS = Transcranial Direct Current Stimulation, AP = Acupuncture, UCSM = Upper Cervical Spine Mobilization, AI = Acupoint Injection, ES = Effortful Swallowing, SSE = Swallowing Strengthening Exercises, ET = Eating Training, FOIS = Functional Oral Intake Scale, VFSS = Videofluoroscopic Swallowing Study, PAS = Penetration–aspiration scale, FDS = Functional Dysphagia Scale, VDS = Video Fluoroscopic Dysphagia Scale, GUSS = Gugging Swallowing Screen, DSRS = Dysphagia Severity Rating Scale, EAT-10 = Eating Assessment Tool-10, SWAL-QOL = Swallow Quality of Life Questionnaire, VRQOL = Voice-related Quality of Life questionnaire, SSA = Standardized Swallowing Assessment, CE = Clinical Efficacy, SDSS = Swallowing Difficulty Scale Score, NTRR = Nasogastric Tube Removal Rate, CCFT = Craniocervical Flexion Test, CVA = Craniovertebral Angle, WST = Water Swallowing Test, FEES = Fiber Optic Endoscopic Evaluation of Swallowing, DREP = Dysphagia Risk Evaluation Protocol, PS = Pooling Score, OTT = Oral Transit Time, PDT = Pharyngeal Delay Time, PTT = Pharyngeal Transit Time, sEMG = Surface Electromyography, MMSE = Mini-Mental State Examination, DOSS = Dysphagia Outcome and Severity Scale, SAP = StrokeAssociated Pneumonia.

A full list of abbreviations and definitions are presented in Supplementary Table 2.

### 3.3 Risk of bias

**Figure 2:**
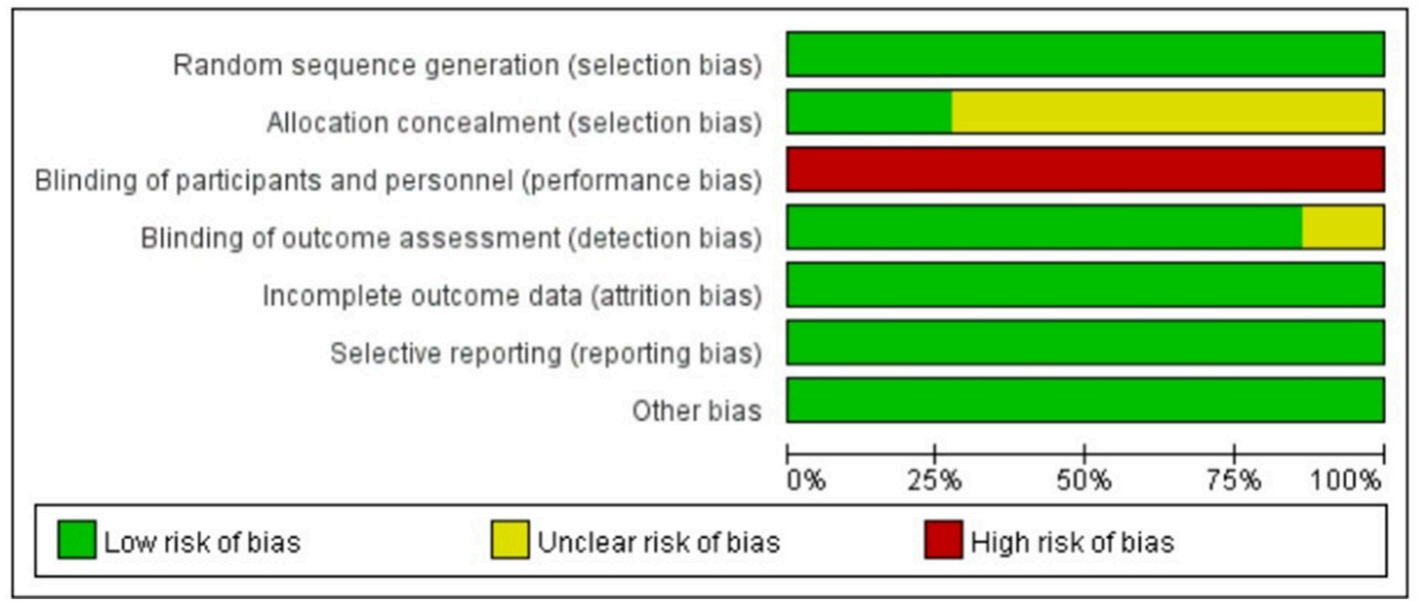
Risk of bias graph.

**Figure 3:**
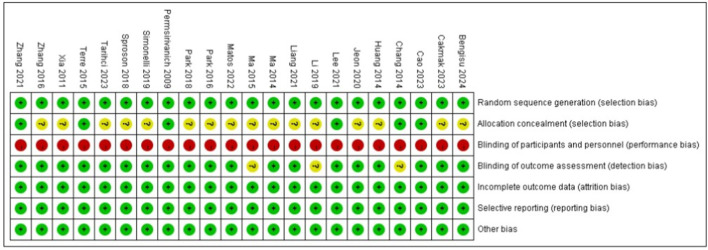
Risk of bias summary.

The methodological quality assessment, as illustrated in Figures 2, 3, reveals that all included trials were rated as low risk in terms of random sequence generation. However, only 6 studies (7, 8, 18, 21–23) provided detailed allocation concealment methods and were consequently rated as low risk, while the remaining 16 studies (2, 5, 6, 9–17, 19, 20, 24) were categorized as unclear due to insufficient information. Regarding blinding procedures, all studies were rated as high risk for both participant and personnel blinding, as the nature of neuromuscular electrical stimulation (NMES) intervention makes complete blinding impractical. In contrast, 21 studies (5–7, 9–11, 13–21) were assessed as low risk for outcome assessment blinding, with 3 studies (2, 8, 12) rated as unclear due to lack of reporting on blinding procedures. Notably, none of the included studies exhibited incomplete outcome data, selective reporting, or other forms of bias.

### 3.4 Meta-analysis

#### 3.4.1 Primary outcome

##### 3.4.1.1 Clinical efficacy

**Figure 4:**
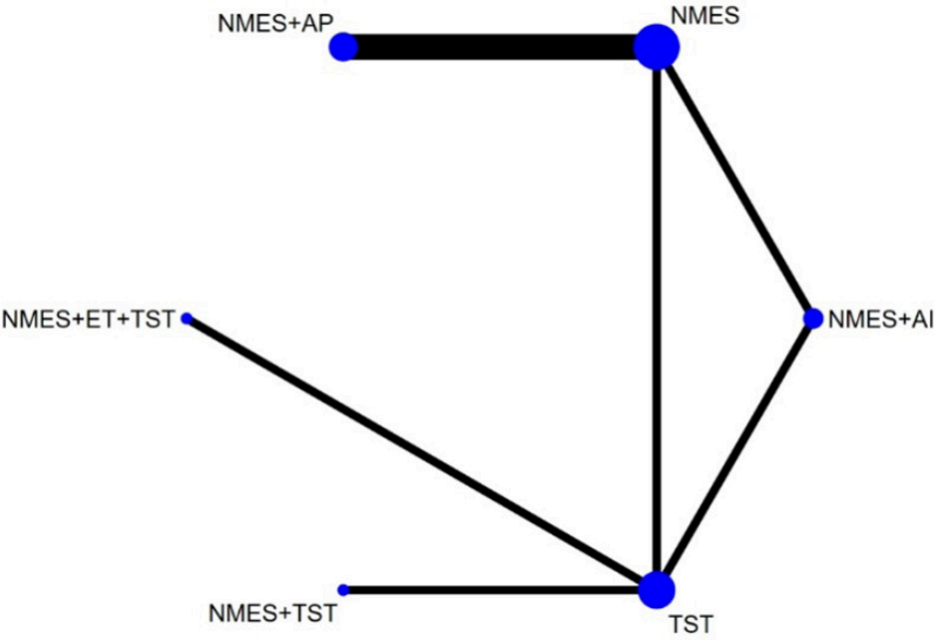
Network plot of clinical efficacy.

**Figure 5:**
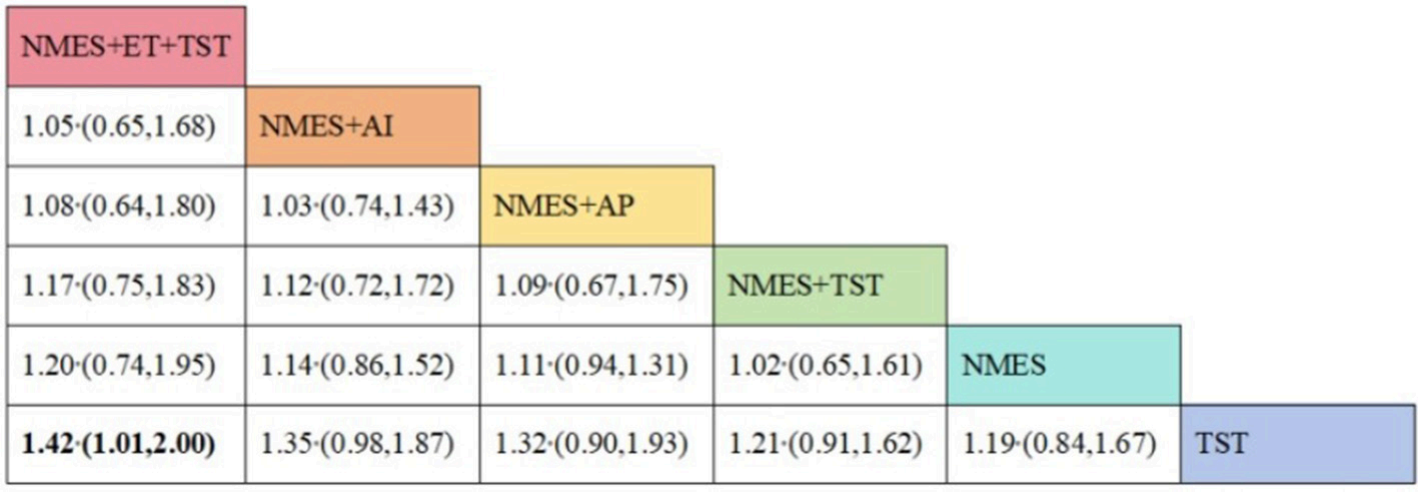
The league figure of clinical efficacy.

**Figure 6:**
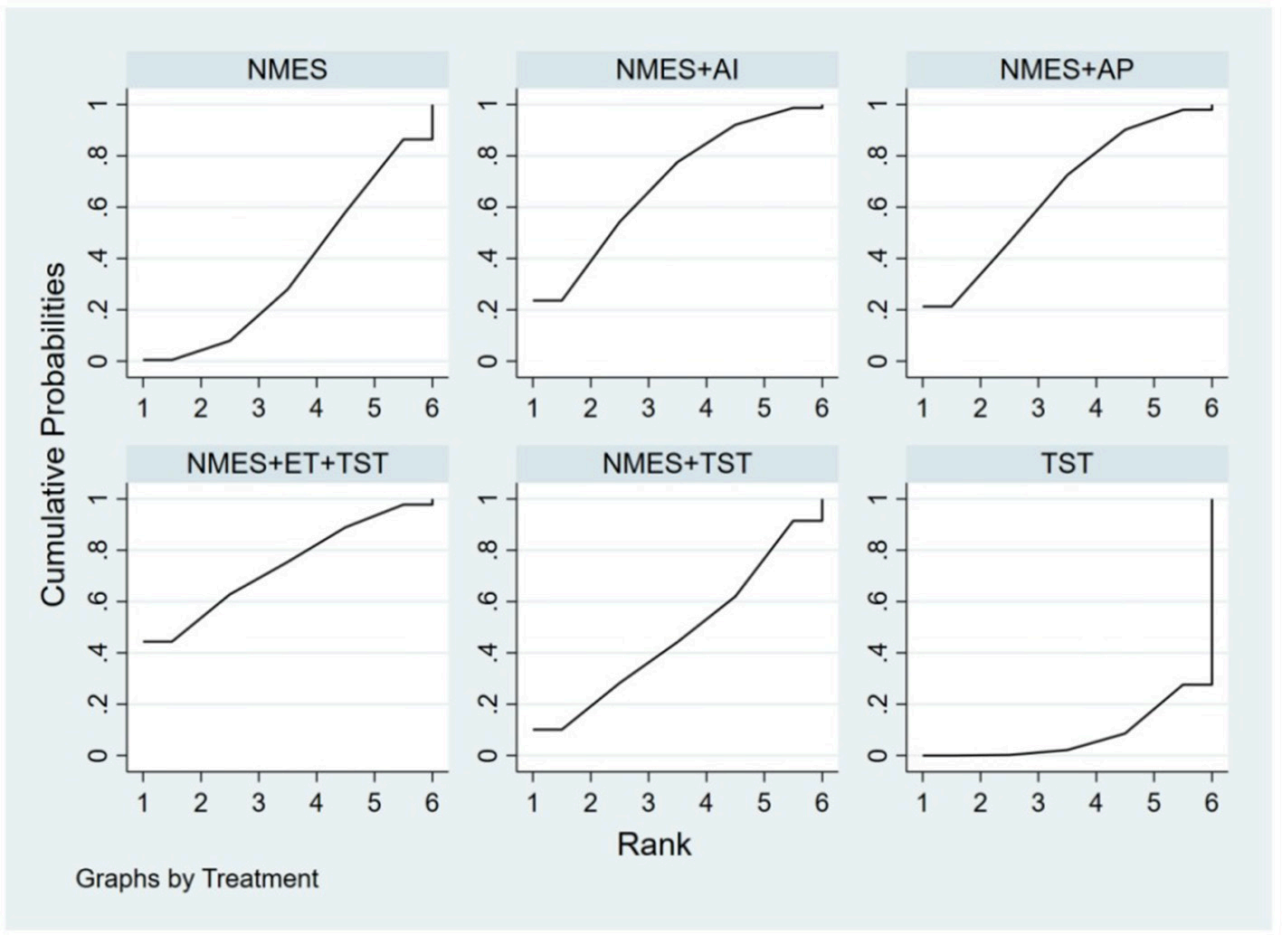
SUCRA probability ranking of clinical efficacy.

**Figure 7:**
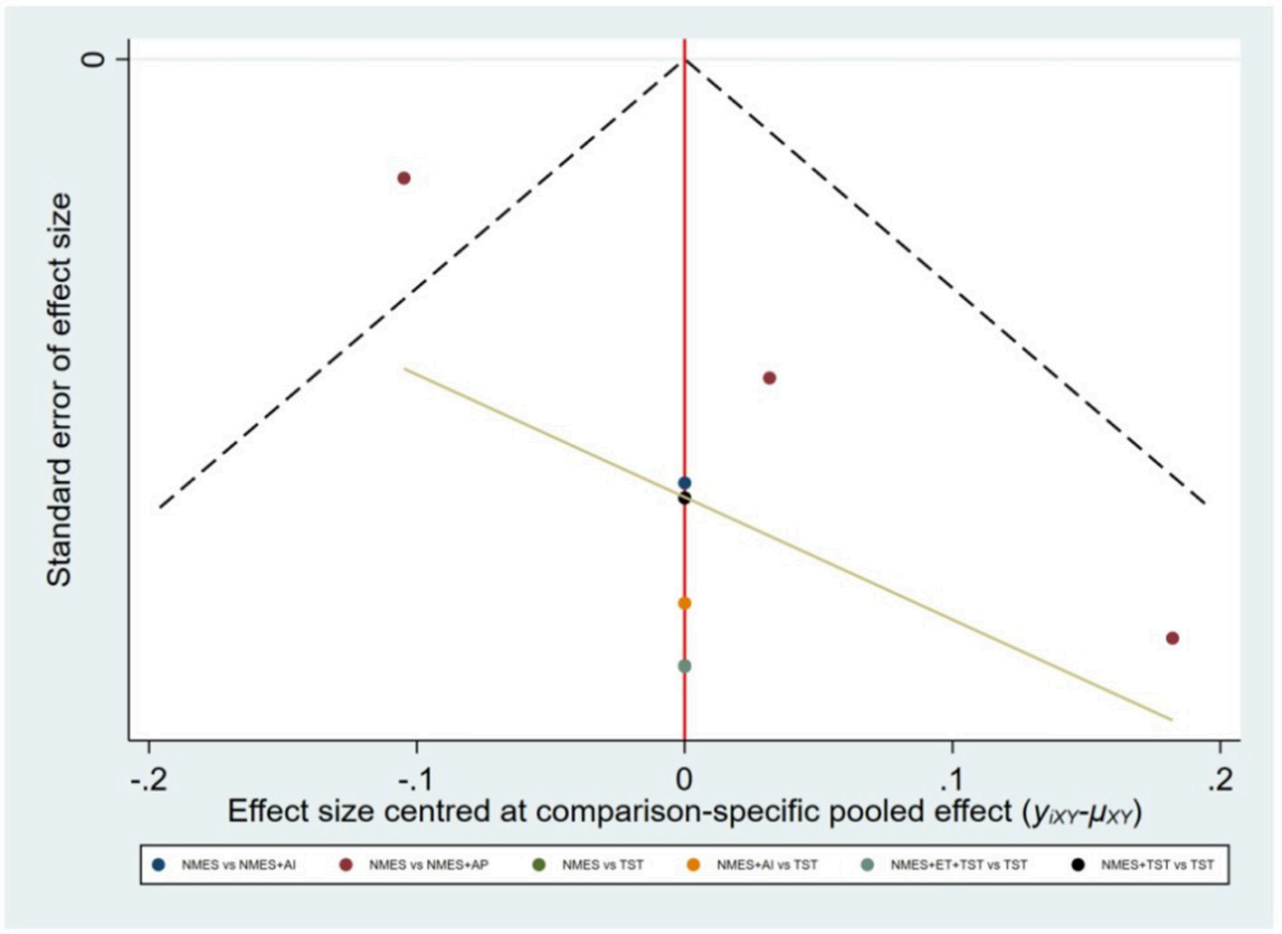
Comparative-corrected funnel plot of clinical efficacy.

Six studies (7, 8, 10–12, 21) reported the clinical efficacy (CE), involving six different interventions. The network plot indicated that the most frequent comparison was between NMES alone and NMES combined with AP, forming a closed loop (Figure 4). The inconsistency model test yielded a *P*-value of 0.19 (*P* > 0.05), suggesting no significant inconsistency. Local inconsistency was assessed using the node-splitting method; however, due to the incomplete network structure, the inconsistency between NMES and NMES+AP could not be evaluated. Other pairwise comparisons revealed no significant differences between direct and indirect comparisons (*P* > 0.05). The league table demonstrated that, compared to TST, NMES combined with ET and TST (1.42, 95% CI: 1.01, 2.00) showed statistically significant improvement in clinical efficacy for patients with neurogenic dysphagia, while NMES+AI (1.35, 95% CI: 0.98, 1.87), NMES+AP (1.32, 95% CI: 0.90, 1.93), NMES+TST (1.21, 95% CI: 0.91, 1.62), and NMES alone (1.19, 95% CI: 0.84, 1.67) exhibited no significant therapeutic differences (Figure 5). Based on SUCRA values, the ranking of interventions from highest to lowest in terms of clinical effective rate was: NMES+ET+TST (73.9%) > NMES+AI (69.3%) > NMES+AP (65.7%) > NMES+TST (47.2%) > NMES (36.2%) > TST (7.7%), indicating that the NMES+ET+TST regimen was the most effective in improving the clinical effective rate for patients with neurogenic dysphagia (Figure 6). The funnel plot for CE suggested potential publication bias, warranting cautious interpretation (Figure 7).

##### 3.4.1.2 FOIS

**Figure 8:**
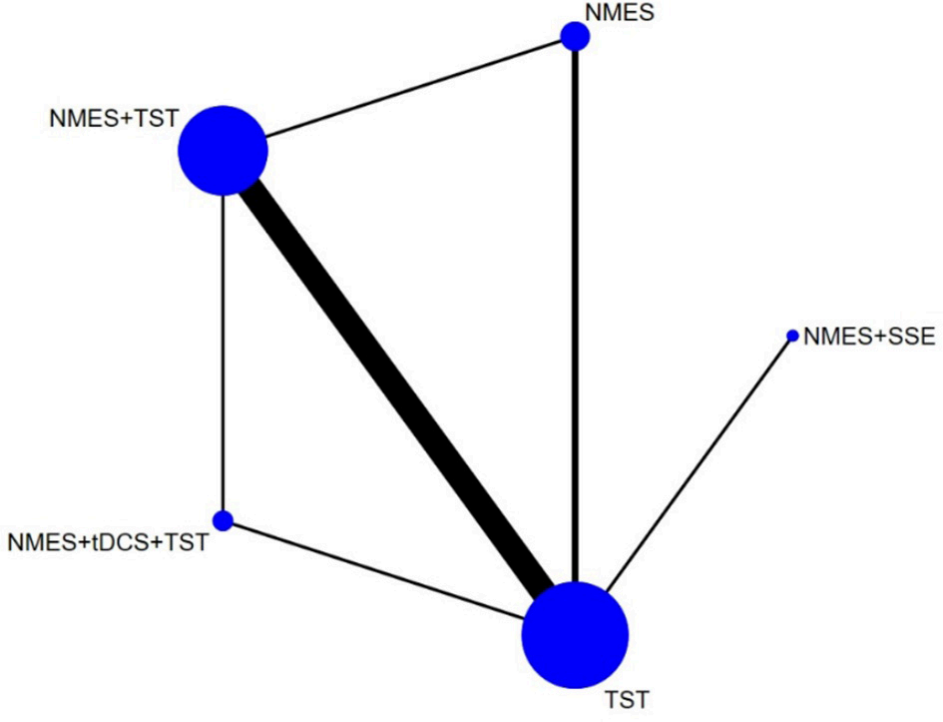
Network plot of FOIS.

**Figure 9:**
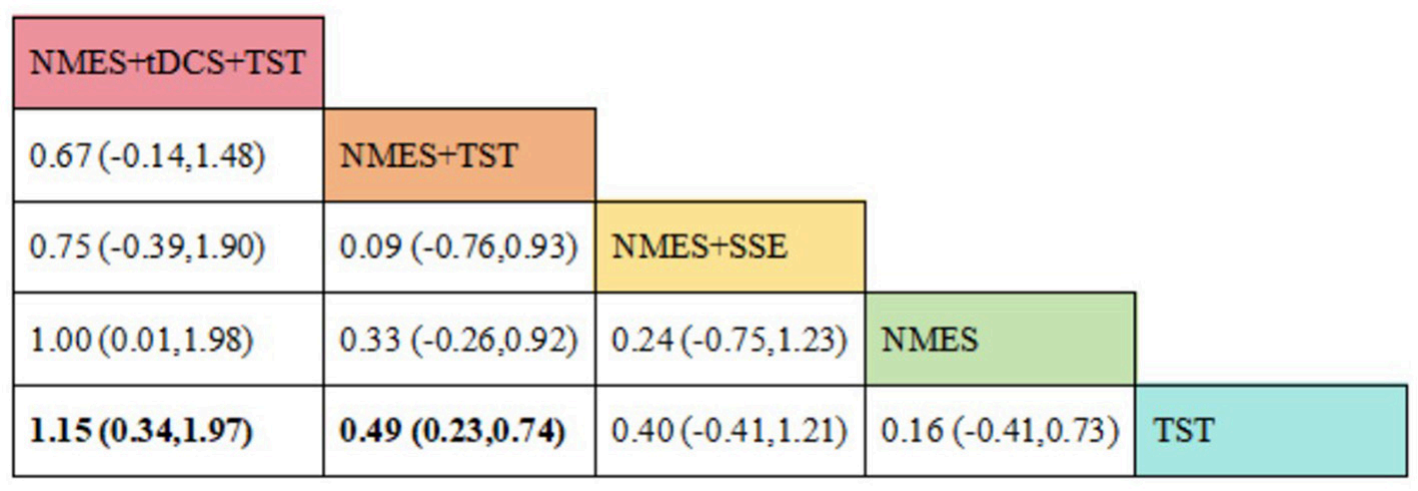
The league figure of FOIS.

**Figure 10:**
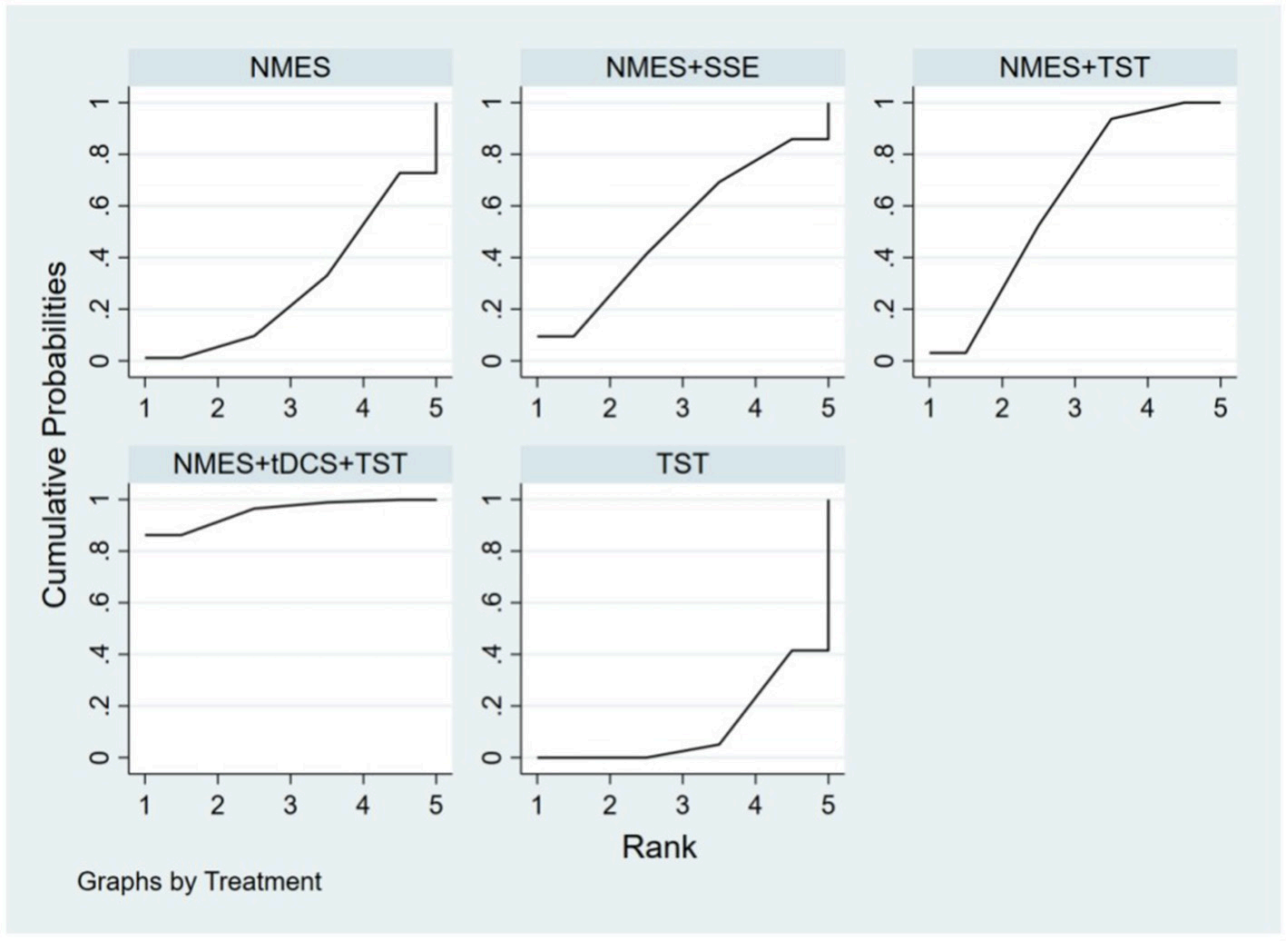
SUCRA probability ranking of FOIS.

**Figure 11:**
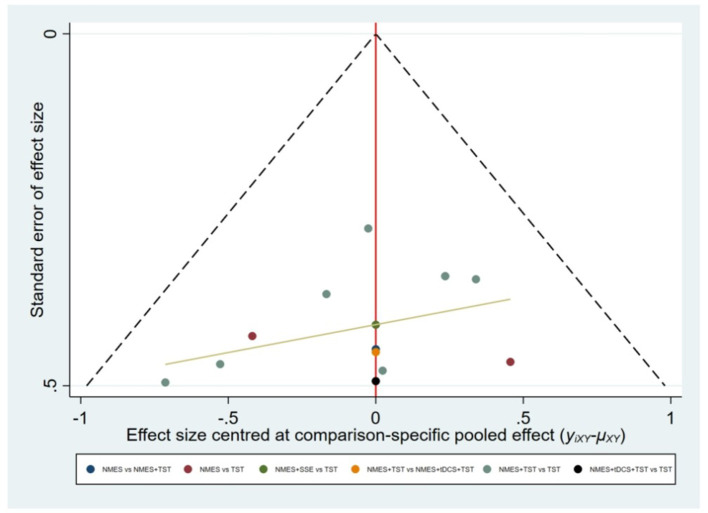
Comparative-corrected funnel plot of FOIS.

Ten RCTs (5, 6, 13, 16–18, 20, 23, 24) reported FOIS scores, encompassing five distinct interventions. The network plot indicated that the comparison between NMES combined with TST and TST alone was the most frequently investigated. Furthermore, the network plot formed two closed loops, as illustrated in Figure 8. Upon loop formation, initial assessment using an inconsistency model for FOIS scores yielded a *P*-value of 0.314 (*P* > 0.05). Subsequently, node-splitting analysis revealed no significant differences between direct and indirect comparisons across all nodes (*P* > 0.05), indicating good consistency between direct and indirect evidence. The league table suggested that, compared to TST alone, NMES combined with tDCS and TST showed a significant advantage in improving FOIS scores (1.15, 95% CI: 0.34, 1.97), as did NMES combined with TST (0.49, 95% CI: 0.23, 0.74). However, NMES combined with SSE (0.40, 95% CI: −0.41, 1.21) and NMES alone (0.16, 95% CI: −0.41, 0.73) demonstrated no significant therapeutic differences (Figure 9). The ranking of interventions based on SUCRA values for FOIS scores, from highest to lowest, was as follows: NMES+tDCS+TST (95.3%) > NMES+TST (62.4%) > NMES+SSE (51.5%) > NMES (29.2%) > TST (11.6%), suggesting that NMES+tDCS+TST appears to be the optimal combination for enhancing FOIS scores and improving swallowing function (Figure 10). Finally, the funnel plot for FOIS scores demonstrated good overall symmetry, indicating a low likelihood of publication bias in the included studies (Figure 11).

##### 3.4.1.3 SSA

**Figure 12:**
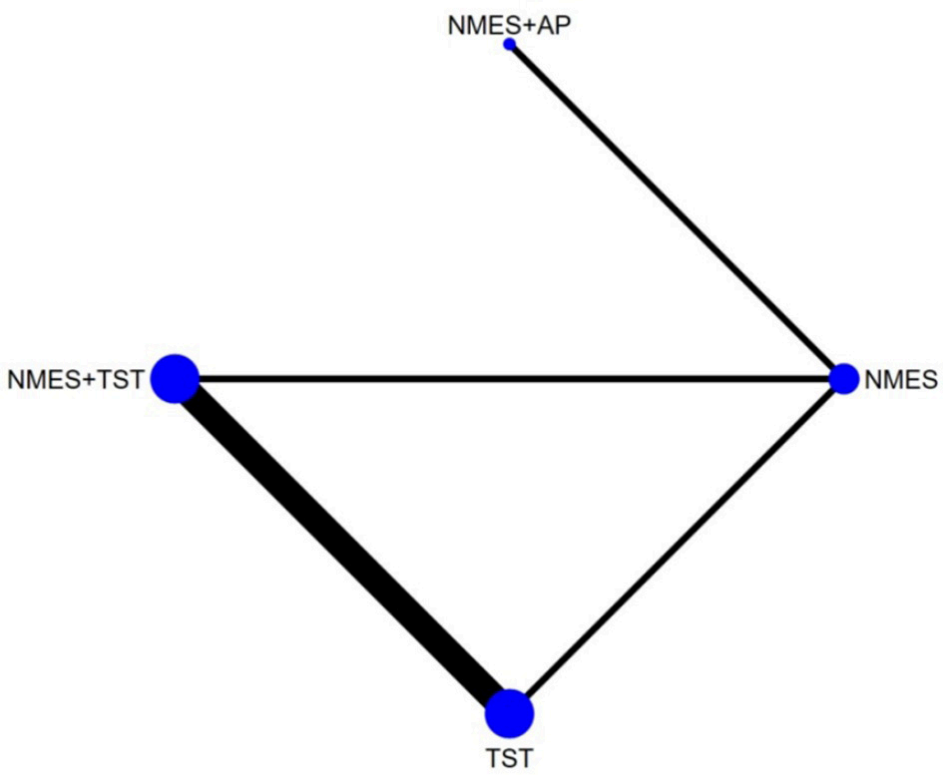
Network plot of SSA.

**Figure 13:**
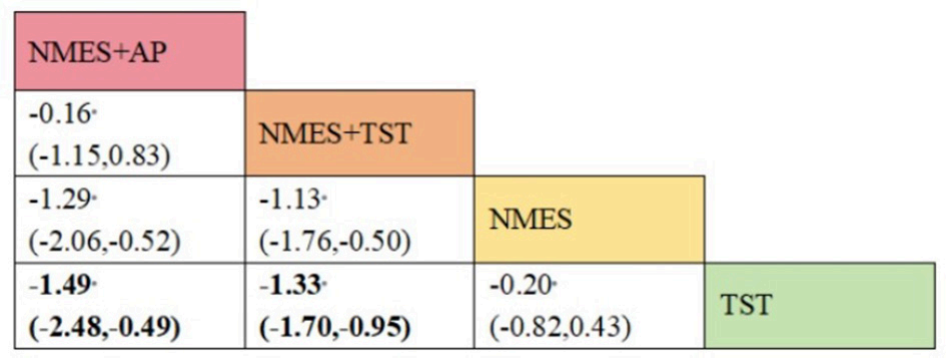
The league figure of SSA.

**Figure 14:**
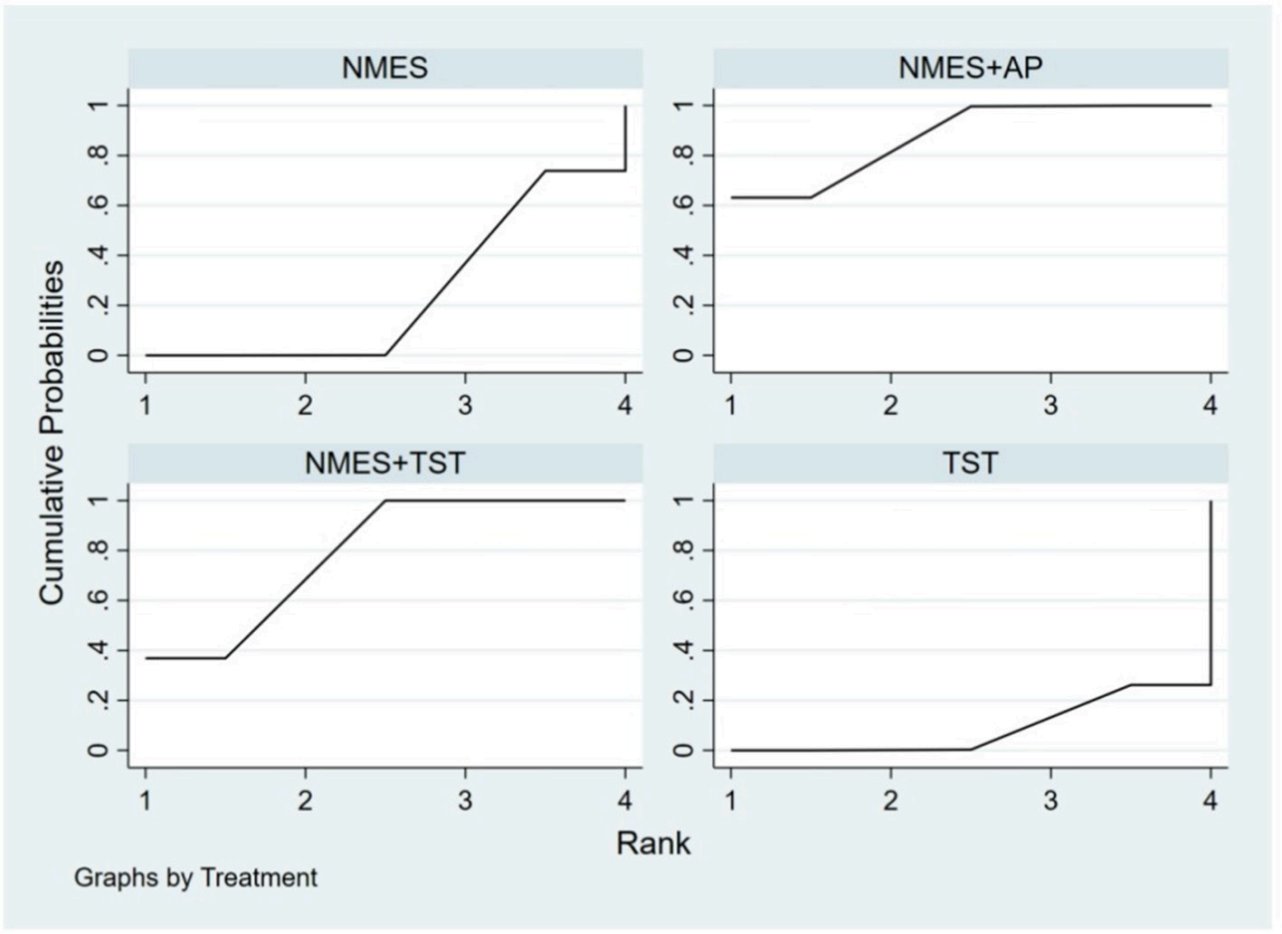
SUCRA probability ranking of SSA.

**Figure 15:**
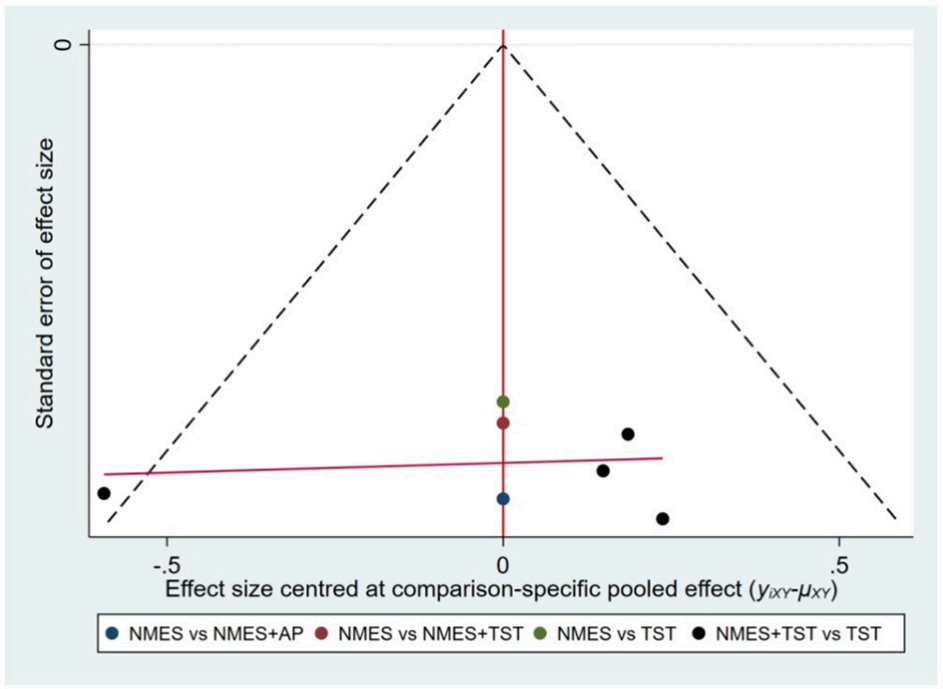
Comparative-corrected funnel plot of SSA.

Five studies (2, 7, 10, 19, 20) reported the standardized swallowing assessment scores, encompassing four distinct interventions. The network plot revealed that the most frequent comparison was between TST alone and NMES combined with TST, forming a closed loop (Figure 12). The inconsistency model test yielded a *P*-value of 0.61 (*P* > 0.05), indicating no significant inconsistency. Local inconsistency was assessed using the node-splitting method; however, due to insufficient data or an incomplete network structure, comparisons between NMES and NMES+AP, as well as NMES+TST and TST, were not available. Other pairwise comparisons showed no significant differences between direct and indirect comparisons (*P* > 0.05). The league table suggested that, compared to TST, NMES+AP (−1.49, 95% CI: −2.48, −0.49) and NMES+TST (−1.33, 95% CI: −1.70, −0.95) demonstrated superior efficacy in reducing SSA scores and improving swallowing function, whereas NMES (−0.20, 95% CI: −0.82, 0.43) showed no therapeutic difference (Figure 13). Based on SUCRA values, the ranking of interventions from highest to lowest was: NMES+AP (87.6%) > NMES+TST (79.0%) > NMES (24.6%) > TST (8.8%), suggesting that NMES+AP may offer significant advantages in improving swallowing function (Figure 14). The funnel plot for SSA indicated potential publication bias (Figure 15).

#### 3.4.2 Secondary outcome

##### 3.4.2.1 PAS

**Figure 16:**
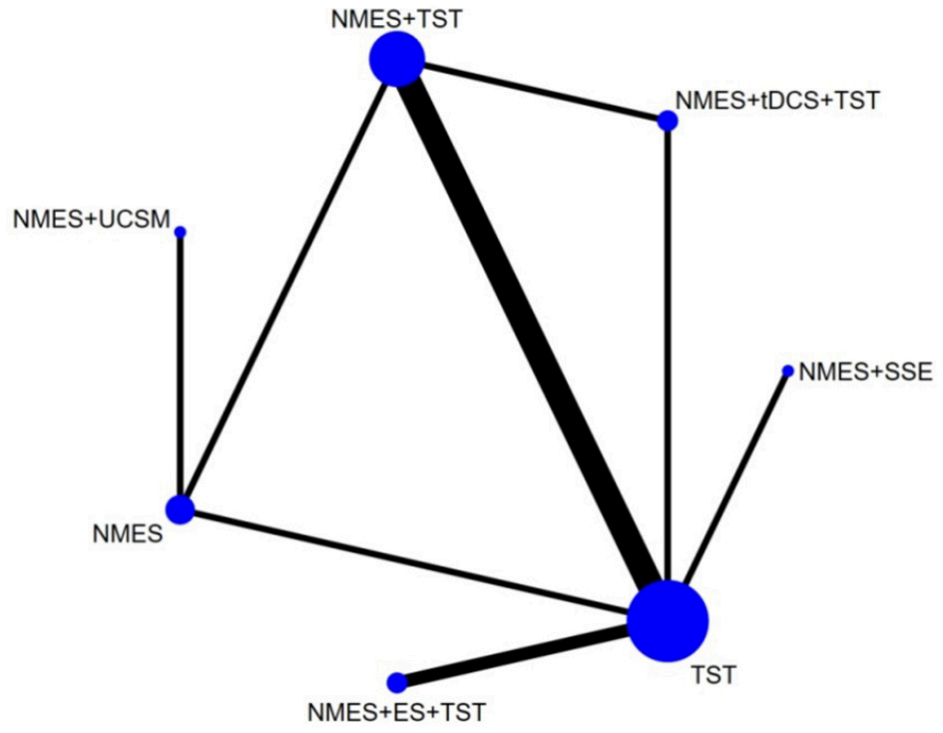
Network plot of PAS.

**Figure 17:**
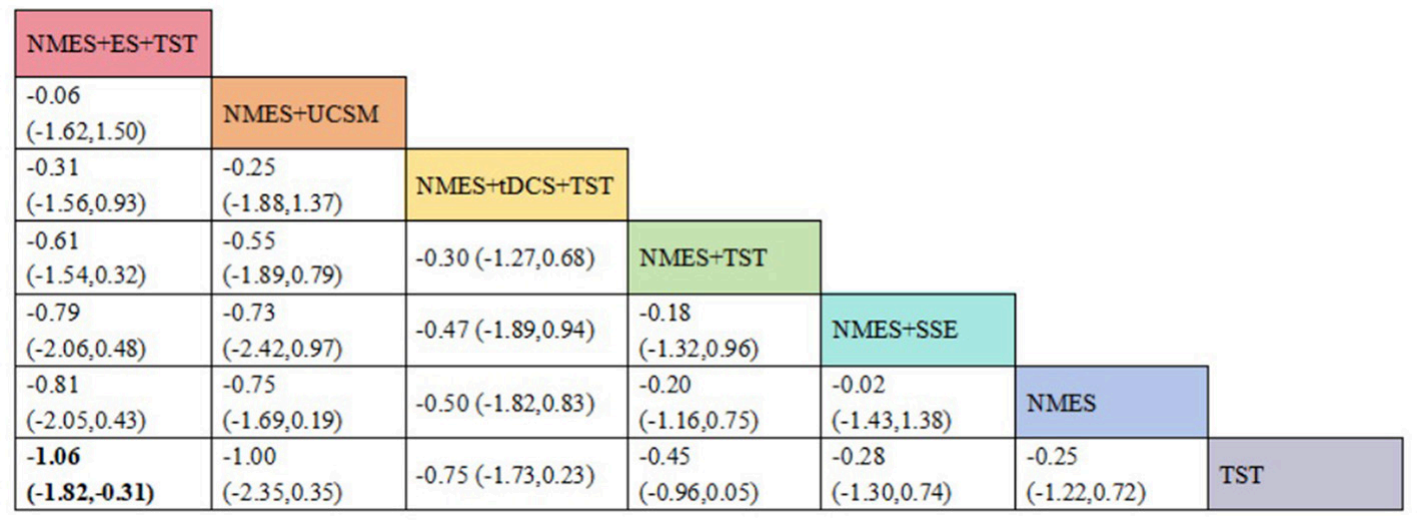
The league figure of PAS.

**Figure 18:**
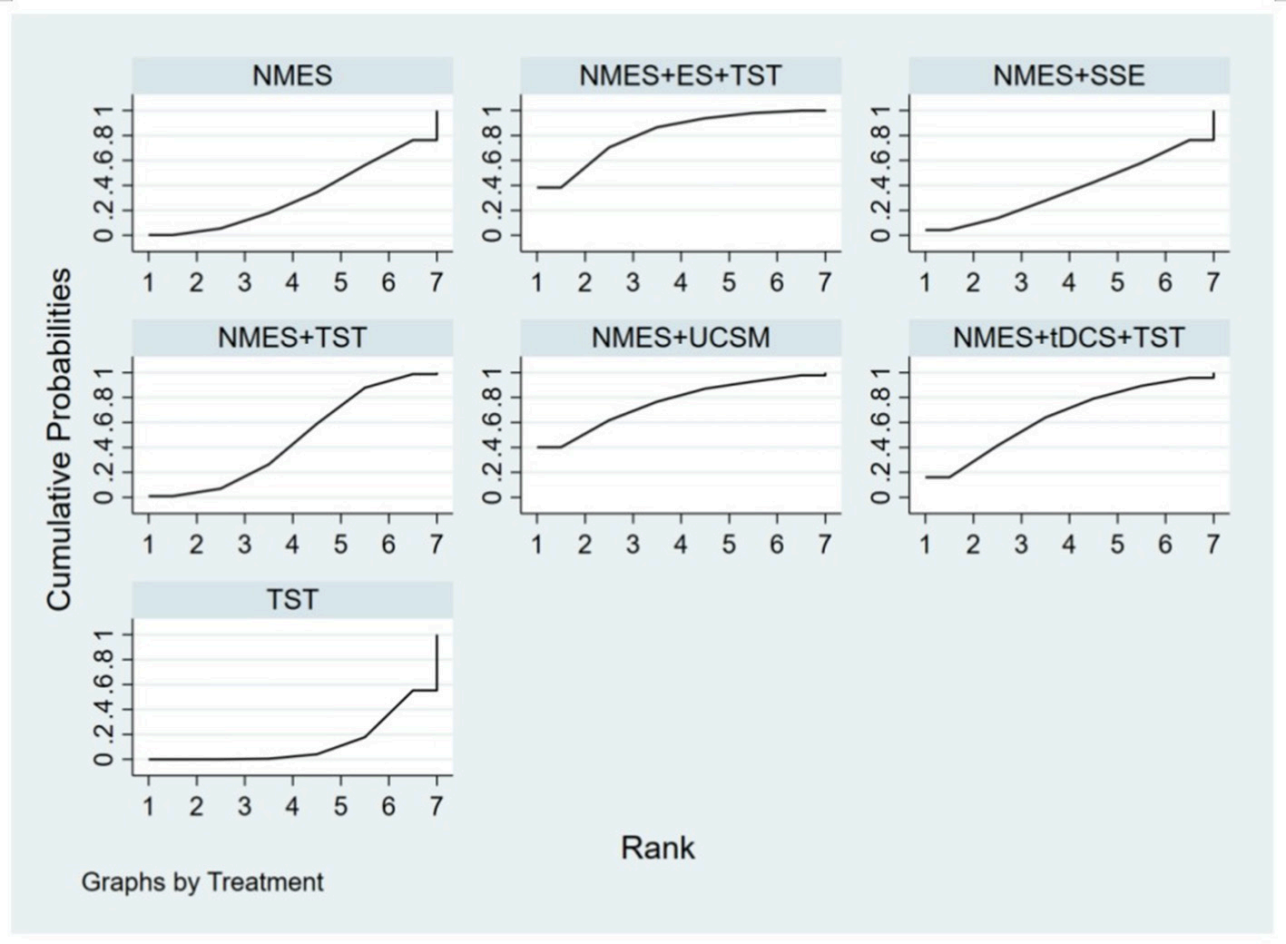
SUCRA probability ranking of PAS.

**Figure 19:**
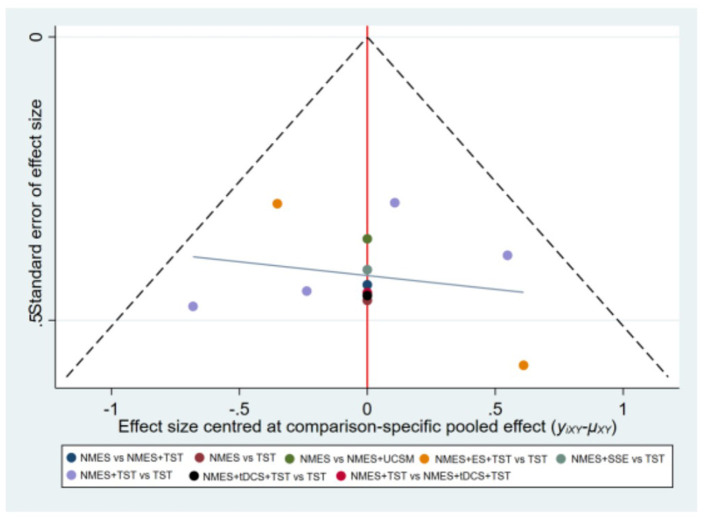
Comparative-corrected funnel plot of PAS.

Eight studies (5, 9, 14–17, 22, 24) reported Penetration-Aspiration Scale scores, involving seven distinct interventions. The network plot revealed that the comparison between TST alone and NMES combined with TST was the most frequently investigated, forming two closed loops (Figure 16). The inconsistency model test yielded a *P*-value of 0.25 (*P* > 0.05), indicating no significant inconsistency. Subsequent local inconsistency assessment using the node-splitting method showed no available data for the comparison between NMES and NMES combined with UCSM, potentially due to insufficient data or an incomplete network structure. For the remaining nodes, no significant differences were observed between direct and indirect comparisons (*P* > 0.05). The league table demonstrated that, compared to TST alone, NMES combined with ES and TST showed a significant advantage in reducing PAS scores and improving aspiration prevention (−1.06, 95% CI: −1.82, −0.31). In contrast, NMES+UCSM (−1.00, 95% CI: −2.35, 0.35), NMES+tDCS+TST (−0.75, 95% CI: −1.73, 0.23), NMES+TST (−0.45, 95% CI: −0.96, 0.05), NMES+Swallowing Strengthening Exercises (SSE) (−0.28, 95% CI: −1.30, 0.74), and NMES alone (−0.25, 95% CI: −1.22, 0.72) showed no significant therapeutic differences (Figure 17). The ranking of interventions based on SUCRA values for PAS scores, from highest to lowest, was as follows: NMES+ES+TST (81.2%) > NMES+UCSM (76.0%) > NMES+tDCS+TST (64.3%) > NMES+TST (46.6%) > NMES+SSE (37.1%) > NMES (31.8%) > TST (12.9%), suggesting that NMES+ES+TST may be the most effective combination for reducing aspiration risk and enhancing swallowing safety (Figure 18). The funnel plot for PAS scores exhibited good symmetry, indicating a low likelihood of publication bias (Figure 19).

## 4 Discussion

Neurogenic dysphagia, resulting from neurological disorders including stroke and Parkinson's disease, involves central or peripheral nerve damage, neurotransmitter imbalance, impaired swallowing reflex pathways, and reduced muscular coordination. These pathophysiological changes primarily affect the oral, pharyngeal, and esophageal phases of swallowing. In the oral phase, common impairments include difficulty forming a bolus, food residue retention, and delayed oral transit time. The pharyngeal phase often presents difficulties such as impaired triggering of the swallowing reflex, delayed pharyngeal clearance, and increased swallowing attempts per bolus. Esophageal phase dysfunction is frequently characterized by esophageal motility disorders, such as lower esophageal sphincter dysfunction, impaired peristalsis, and esophageal obstruction, which can lead to symptoms like regurgitation, retrosternal discomfort, and aspiration (25). These dysphagic symptoms can predispose patients to serious complications including choking, aspiration pneumonia, and malnutrition, severely compromising their quality of life (26). Among various therapeutic interventions for dysphagia, NMES has emerged as a promising therapeutic modality. NMES facilitates the restoration of swallowing function through external electrical stimulation that activates axonal motor nerve terminals and muscle fibers, thereby promoting central nervous system recovery and strengthening impaired oropharyngeal musculature (27).

This study represents the first comprehensive network meta-analysis to systematically comparing the therapeutic efficacy of NMES combined with various interventions (including NMES+TST, NMES+tDCS+TST, NMES+AP, NMES+AI, NMES+UCSM, NMES+ES+TST, and NMES+ET+TST), standalone NMES and TST in the management of neurogenic dysphagia. The primary outcome measures included the FOIS, SSA, and clinical efficacy, with the PAS serving as a secondary outcome.

Our findings demonstrate that both combined NMES therapies, standalone NMES and TST are effective in improving swallowing function and reducing aspiration, but NMES combined with other therapies generally exhibiting superior clinical efficacy over either standalone NMES or TST. According to the efficacy ranking, NMES+tDCS+TST emerged as the most effective combination for enhancing FOIS scores. The therapeutic mechanism of tDCS involves delivering weak direct currents to the cerebral cortex through electrodes placed over targeted cortical areas, modulating brain function to enhance cortical excitability and neuroplasticity (28). Evidence suggests that tDCS particularly improves motor function in patients with post-stroke dysphagia, with anodal tDCS application over the pharyngeal motor cortex enhancing excitability to facilitate dysphagia recovery (29).

The combination of tDCS and NMES creates a synergistic effect by augmenting the brain's capacity to integrate sensory and motor inputs, promoting neuroplasticity at both cortical and peripheral levels (30). This dual-level approach enhances muscular strength while the incorporation of TST provides additional sensory feedback, improving coordination and establishing a comprehensive rehabilitation strategy.

Our findings align with and extend the current evidence base in neurogenic dysphagia management. The recent European Stroke Organization and European Society for Swallowing Disorders (ESO–ESSD) guideline (27) recognizes the potential of neuromodulation techniques like tDCS and peripheral stimulation, while underscoring the need for more robust evidence regarding their optimal application and combination. A systematic review by Wang et al. (4) concluded that transcutaneous neuromuscular electrical stimulation is a beneficial adjunctive therapy for post-stroke dysphagia, which is consistent with our observation that NMES-based therapies generally outperform traditional training alone. Furthermore, a broader literature review by Panebianco et al. (1) highlighted the complex pathophysiology of neurogenic dysphagia and the importance of multimodal interventions. Our network meta-analysis directly addresses these calls by providing a hierarchical comparison of various combination strategies, thereby offering preliminary evidence on which central-peripheral integrated approach might be most efficacious for specific swallowing outcomes.

The SSA score serves as a validated and reliable tool for evaluating swallowing function. Our findings demonstrate that the combination of NMES+AP exhibits superior efficacy in reducing SSA scores compared to both NMES+TST and standalone NMES or TST interventions. Acupuncture, an ancient Chinese medical technique, exerts its therapeutic effects through stimulation of specific acupoints, modulating the functional activities of both the central and autonomic nervous systems. This modulation enhances excitability in the swallowing centers, facilitates neural pathway restoration, and promotes circulatory improvement and tissue functional recovery (31–33). The therapeutic synergy between NMES and acupuncture can be attributed to their complementary mechanisms of action. While NMES directly enhances muscular function through peripheral nerve stimulation, acupuncture improves neural control via central nervous system regulation. This combined approach achieves dual modulation at both peripheral and central levels, synergistically promoting neuroplasticity. Consequently, this integrated therapeutic strategy results in more comprehensive improvement of swallowing function, as evidenced by the significant reduction in SSA scores.

The PAS serves as a standardized instrument for assessing the severity of aspiration. Our analysis revealed that the combined therapeutic regimen of NMES+TST+ES demonstrated significant superiority in reducing PAS scores. Effortful swallowing, a therapeutic maneuver, has been shown to effectively enhance hyoid bone movement and induce robust activation of hyoid musculature within a relatively short duration (34, 35). Training of the suprahyoid muscle group directly reduces aspiration risk and influences protective mechanisms during swallowing (36). The incorporation of effortful swallowing into NMES combined with TST creates a synergistic effect by further strengthening pharyngeal musculature and optimizing the timing of the swallowing reflex. This multimodal approach enhances both the sensitivity and coordination of the swallowing reflex, leading to significant reductions in PAS scores. Consequently, this therapeutic strategy effectively mitigates the risk of food or liquid entry into the airway and decreases the incidence of aspiration pneumonia (37).

Through this study, we conclude that central-peripheral combined stimulation therapy appears to be most effective treatment strategy. This is substantiated by the consistent superiority of combinations that simultaneously target both the central and peripheral nervous systems across primary and secondary outcomes. Specifically, the highest-ranked intervention for improving FOIS scores was NMES+tDCS+TST, where tDCS modulates cortical excitability and neuroplasticity centrally, while NMES acts peripherally to strengthen oropharyngeal musculature. Similarly, for SSA scores, the most effective regimen was NMES+AP, which integrates peripheral electrical stimulation with central neuromodulation via acupuncture. Furthermore, the combination of NMES+ES+TST, which ranked first for reducing PAS scores, incorporates a volitional central maneuver (effortful swallowing) to enhance the efficacy of peripheral stimulation and traditional training. These findings indicate that the top-performing interventions for each key outcome measure all embody the therapeutic principle of central-peripheral integration.

In summary, the combination of NMES, which targets the peripheral nervous system, with other therapies that act on the central nervous system—referred to as combined central-peripheral stimulation—demonstrates significant advantages in improving swallowing function, reducing aspiration risk, and enhancing swallowing safety. This integrated approach may be considered a preferred therapeutic strategy for the management of neurogenic dysphagia.

## 5 Study limitations

Several limitations should be acknowledged in the present study. First, substantial heterogeneity was observed across included studies regarding NMES parameters (including treatment frequency, duration, intensity, and repetition), patient characteristics (such as underlying etiology, site of neural injury, and disease duration), intervention protocols, and outcome measurement tools. Second, the relatively small sample sizes in some studies may have compromised statistical power to detect significant differences. Due to the limited number of studies and insufficient data, we did not perform subgroup analyses. Third, the observed funnel plot asymmetry suggests potential publication bias, which may affect the robustness of our findings. Fifth, regarding the outcome of CE, although it was defined based on significant improvement according to the original authors' criteria in the included studies, we recognize that the specific operational definitions and assessment criteria for CE varied across trials. This variability, which could include differences in the choice of assessment tools like FOIS, SSA or the thresholds for “improvement”, may introduce heterogeneity and potentially bias the pooled estimate for this outcome. The interpretation of the findings for CE should therefore consider this limitation. It is worth noting that most of the included studies lacked blinding, and the inclusion of only English and Chinese language sources may introduce certain biases.

Future research should focus on standardizing both NMES protocols and traditional swallowing training regimens to minimize heterogeneity and enhance comparability across studies. Additionally, etiology-based subgroup analyses could help identify patient populations that derive maximal benefit from specific interventions. However, current research predominantly focuses on post-stroke dysphagia, with limited evidence available for neurogenic dysphagia resulting from other causes. Therefore, large-scale, high-quality randomized controlled trials involving diverse patient populations are warranted to strengthen the evidence base.

## 6 Conclusions

This network meta-analysis demonstrates that NMES combination therapy is superior to NMES or TST alone. The ranking plot suggests that central-peripheral combined stimulation therapy appears to be the most effective treatment for improving swallowing function and reducing aspiration rates. However, the funnel plot for some outcome indicators suggests potential bias, necessitating cautious interpretation of these findings.

## Data Availability

The original contributions presented in the study are included in the article/Supplementary material, further inquiries can be directed to the corresponding author.
