# Supplementary material for: Comparative efficacy of combined and single neuromuscular electrical stimulation and traditional swallowing training for neurogenic dysphagia: a network meta-analysis
Source: Front Neurol. 2025 Dec 3;16:1700317. doi: 10.3389/fneur.2025.1700317 (PMC12709119; doi:10.3389/fneur.2025.1700317)
Supplement: Supplementary file 1 [file Table_1.docx]

**Supplementary table 1**. The literature search strategy

PubMed

("dysphagia" OR ((((((((Deglutition Disorders) OR (Deglutition Disorder)) OR (Disorders, Deglutition)) OR (Swallowing Disorders)) OR (Swallowing Disorder)) OR (Oropharyngeal Dysphagia)) OR (Dysphagia, Oropharyngeal)) OR (Esophageal Dysphagia)) OR (Dysphagia, Esophageal)) AND (“Neuromuscular Electrical Stimulation” OR (((NMES) OR (Electrical muscle stimulation)) OR (Electrostimulation)) OR (Neuromuscular stimulation)) AND ((((randomized controlled trial) OR (Randomized)) OR (Placebo)) OR (RCT))

Embase

('dysphagia':ti OR 'Deglutition Disorders':ti OR 'Deglutition Disorder':ti OR 'Disorders, Deglutition':ti OR 'Swallowing Disorders':ti OR 'Swallowing Disorder':ti OR 'Oropharyngeal Dysphagia':ti OR 'Dysphagia, Oropharyngeal':ti OR 'Esophageal Dysphagia':ti OR 'Dysphagia, Esophageal':ti) AND ('Neuromuscular Electrical Stimulation':ti OR 'NMES':ti OR 'Electrical muscle stimulation':ti OR 'Electrostimulation':ti OR 'Neuromuscular stimulation':ti )

Web of Science

TI= (dysphagia OR Deglutition Disorders OR Deglutition Disorder OR Disorders, Deglutition OR Swallowing Disorders OR Swallowing Disorder OR Oropharyngeal Dysphagia OR Dysphagia, Oropharyngeal OR Esophageal Dysphagia OR Dysphagia, Esophageal)) AND TI=(Neuromuscular Electrical Stimulation OR NMES OR Electrical muscle stimulation OR Electrostimulation OR Neuromuscular stimulation)

The Cochrane Library

(dysphagia OR Deglutition Disorders OR Deglutition Disorder OR Disorders, Deglutition OR Swallowing Disorders OR Swallowing Disorder OR Oropharyngeal Dysphagia OR Dysphagia, Oropharyngeal OR Esophageal Dysphagia OR Dysphagia, Esophageal)) AND (Neuromuscular Electrical Stimulation OR NMES OR Electrical muscle stimulation OR Electrostimulation OR Neuromuscular stimulation)

Searching operation

For all databases, we used the related terms or map term to subject heading options to include the terms and all the synonyms automatically.

**Supplementary table 2** List of Abbreviations

| **Abbreviation** | **Full form** |
| --- | --- |
| T | treatment group |
| C | control group |
| M/F | male and female |
| yr | year |
| NMES | Neuromuscular Electrical Stimulation |
| TST | Traditional Swallowing Training |
| tDCS | Transcranial Direct Current Stimulation |
| AP | Acupuncture |
| UCSM | Upper Cervical Spine Mobilization |
| AI | Acupoint Injection |
| ES | Effortful Swallowing |
| SSE | Swallowing Strengthening Exercises |
| ET | Eating Training |
| FOIS | Functional Oral Intake Scale |
| VFSS | Video fluoroscopic Swallowing Study |
| PAS | Penetration–aspiration scale |
| FDS | Functional Dysphagia Scale |
| VDS | Video Fluoroscopic Dysphagia Scale |
| GUSS | Gugging Swallowing Screen |
| DSRS | Dysphagia Severity Rating Scale |
| EAT-10 | Eating Assessment Tool-10 |
| SWAL-QOL | Swallow Quality of Life Questionnaire |
| VRQOL | Voice-related Quality of Life questionnaire |
| SSA | Standardized Swallowing Assessment |
| CE | Clinical Efficacy |
| SDSS | Swallowing Difficulty Scale Score |
| NTRR | Nasogastric Tube Removal Rate |
| CCFT | Craniocervical Flexion Test |
| CVA | Craniovertebral Angle |
| WST | Water Swallowing Test |
| FEES | Fiber Optic Endoscopic Evaluation of Swallowing |
| DREP | Dysphagia Risk Evaluation Protocol |
| PS | Pooling Score |
| OTT | Oral Transit Time |
| PDT | Pharyngeal Delay Time |
| PTT | Pharyngeal Transit Time |
| sEMG | Surface Electromyography |
| MMSE | Mini-Mental State Examination |
| DOSS | Dysphagia Outcome and Severity Scale |
| SAP | Stroke Associated Pneumonia |
